# Immunological correlates of protection afforded by PHV02 live, attenuated recombinant vesicular stomatitis virus vector vaccine against Nipah virus disease

**DOI:** 10.3389/fimmu.2023.1216225

**Published:** 2023-09-04

**Authors:** Thomas P. Monath, Richard Nichols, Friederike Feldmann, Amanda Griffin, Elaine Haddock, Julie Callison, Kimberly Meade-White, Atsushi Okumura, Jamie Lovaglio, Patrick W. Hanley, Chad S. Clancy, Carl Shaia, Wasima Rida, Joan Fusco

**Affiliations:** ^1^ Crozet Biopharma LLC, Lexington, MA, United States; ^2^ Public Health Vaccines Inc., Cambridge, MA, United States; ^3^ Rocky Mountain Veterinary Branch, Division of Intramural Research, National Institute of Allergy and Infectious Diseases, National Institutes of Health, Hamilton, MT, United States; ^4^ Laboratory of Virology, Division of Intramural Studies, National Institute of Allergy and Infectious Diseases, National Institutes of Health, Hamilton, MT, United States; ^5^ Biostatistics Consultant, Arlington, VA, United States

**Keywords:** Nipah virus, vaccine, recombinant VSV, immune correlate, neutralizing antibody

## Abstract

**Introduction:**

Immune correlates of protection afforded by PHV02, a recombinant vesicular stomatitis (rVSV) vector vaccine against Nipah virus (NiV) disease, were investigated in the African green monkey (AGM) model. Neutralizing antibody to NiV has been proposed as the principal mediator of protection against future NiV infection.

**Methods:**

Two approaches were used to determine the correlation between neutralizing antibody levels and outcomes following a severe (1,000 median lethal doses) intranasal/intratracheal (IN/IT) challenge with NiV (Bangladesh): (1) reduction in vaccine dose given 28 days before challenge and (2) challenge during the early phase of the antibody response to the vaccine.

**Results:**

Reduction in vaccine dose to very low levels led to primary vaccine failure rather than a sub-protective level of antibody. All AGMs vaccinated with the nominal clinical dose (2 × 10^7^ pfu) at 21, 14, or 7 days before challenge survived. AGMs vaccinated at 21 days before challenge had neutralizing antibodies (geometric mean titer, 71.3). AGMs vaccinated at 7 or 14 days before challenge had either undetectable or low neutralizing antibody titers pre-challenge but had a rapid rise in titers after challenge that abrogated the NiV infection. A simple logistic regression model of the combined studies was used, in which the sole explanatory variable was pre-challenge neutralizing antibody titers. For a pre-challenge titer of 1:5, the predicted survival probability is 100%. The majority of animals with pre-challenge neutralizing titer of ≥1:20 were protected against pulmonary infiltrates on thoracic radiograms, and a majority of those with titers ≥1:40 were protected against clinical signs of illness and against a ≥fourfold antibody increase following challenge (indicating sterile immunity). Controls receiving rVSV-Ebola vaccine rapidly succumbed to NiV challenge, eliminating the innate immunity stimulated by the rVSV vector as a contributor to survival in monkeys challenged as early as 7 days after vaccination.

**Discussion and conclusion:**

It was concluded that PHV02 vaccine elicited a rapid onset of protection and that any detectable level of neutralizing antibody was a functional immune correlate of survival.

## Introduction

A central concept in vaccinology is the definition of the immune responses provoked by a vaccine and the role of these responses in protecting against the target (infectious) disease (1). Ideally, the immune response can serve as a surrogate for randomized controlled trials (RCTs) since the former generally requires a much smaller sample size, does not require a population of subjects affected by the disease, can be applied to special populations (e.g., the elderly, infants, and diverse ethnic groups), and can answer important questions—in particular, the durability of protection. The majority of existing vaccines appear to protect against future exposure *via* antibodies—in most cases, mechanistically functional antibodies (2). However, from a regulatory perspective, an immune surrogate does not need to be functional and can be a representative predictor or biomarker, signaling that an underlying functional response that is responsible for clinical benefit to the subject has occurred. The latter concept is embodied in the FDA’s “Accelerated Approval” pathway which allows marketing authorization of a vaccine for prevention of a serious condition or for an unmet medical need based on a surrogate endpoint (immunological biomarker) that predicts clinical benefit. The sponsor is required to confirm that there is a meaningful clinical benefit in phase IV efficacy or effectiveness trial post-marketing (3).

Examples of vaccines that have been approved in the US or elsewhere based on an immune surrogate include those for COVID-19, influenza, pneumococcal and meningococcal disease, smallpox, rabies, yellow fever, and Japanese encephalitis, but in most cases, it has been possible to compare the immune response to a pre-existing vaccine with established efficacy or effectiveness. The use of a non-inferiority design is not feasible for vaccines against new target indications without a pre-existing accepted vaccine or when an immunologic correlate has not been defined. In such cases, immune responses in animal disease models where protection can be assessed by experimental challenge are bridged to responses in human vaccine trials. As an example, immune responses to the Ad26 vector prime-MVA-BN-Filo boost vaccine against Ebola virus disease were bridged to human immune responses in clinical trials, showing a close correlation between protection in nonhuman primates and IgG-binding antibody levels (4). Inferences may also be drawn from a comparison of vaccine responses to natural infection immunity (5). Ideally, a level of protective immunity, e.g., an antibody titer, is defined, providing quantitative, statistical means to determine protection based on the surrogate.

NiV disease is a relatively rare but highly lethal bat-borne zoonosis in south and southeast Asia caused by a single-strand, negative-sense RNA virus in the Henipavirus genus, family *Paramyxoviridae.* The disease is characterized by acute, severe pneumonia and encephalitis and has a 40%–75% (or higher) case fatality rate (6). Recrudescent, late encephalitis is described (7), a feature which has implications for vaccine development. The development of NiV vaccines is a high priority for the World Health Organization (WHO) (8) and the Coalition for Epidemic Preparedness Innovations (CEPI) since the virus is transmissible from person to person by the respiratory route and has a pandemic potential. Multiple NiV vaccines are in development, and three have entered phase 1 clinical trials [a subunit protein vaccine (NCT04199169), an mRNA vaccine (NCT05398796), and a recombinant vesicular stomatitis (rVSV)-vectored live, attenuated vaccine (NCT05178901), which is the vaccine candidate described here].

All vaccines against NiV face the same problem for regulatory approval, namely, that (at least in the face of the current epidemiological situation of unpredictable, intermittent small outbreaks) RCTs for efficacy are likely infeasible. The largest outbreak to date occurred in Malaysia (1998–1999), with 265 cases and 105 deaths (9), and subsequent outbreaks have involved a few to tens of cases. This leaves two potential pathways to regulatory authorization: accelerated approval and the Animal Rule (AR). While both pathways require animal data on immune correlates that are then bridged to human vaccine responses, the AR is acknowledged to be difficult and requires a highly stringent understanding of pathogenesis in the animal model. The time required for the latter may not be consistent with current objectives of rapid vaccine development against emerging public health threats (10). The question, therefore, is whether an immune surrogate for protective immunity against NiV that would allow emergency use authorization and, eventually, full marketing approval can be defined.

At this stage of development of NiV vaccines, there are encouraging indications of the feasibility of identifying an immune surrogate of protection. These indications are based on animal model data since there are few asymptomatic human infections and few survivors of the natural disease for comparison of natural vs. artificial (vaccine-induced) immune responses. As for many other vaccines, antibodies are the principal mediator of protection against NiV (11, 12). Protection against challenge in animal models can be passively transferred by serum polyclonal antibodies (13) and by human monoclonal antibodies with neutralizing activity (14), which have been characterized at the epitope level (15–17). Levels of neutralizing antibody required for protection in a mouse model employing NiV pseudovirus challenge have been defined based on both passive transfer of antibody and active immunization (18). The body of evidence indicates that the classical viral neutralization test result may serve as a surrogate for protection.

In this paper, we describe studies of the rVSV-vectored vaccine in an established nonhuman primate model of NiV disease, the African green monkey (AGM) (19), with a principal objective of elucidating an immune correlate of protection. The vaccine is a live, attenuated recombinant vesicular stomatitis (Indiana) virus (rVSV) developed by reverse genetics in which the glycoprotein (G) gene of VSV (the principal neurovirulence gene) has been deleted and replaced with the corresponding envelope glycoprotein genes of both Ebola virus (Kikwit) (EBOV GP) and Nipah virus (Bangladesh) (NiV G). The EBOV GP is required for fusion and cell entry, which are not mediated by the NiV G protein. The G protein responsible for attachment to cell receptors, principally ephrin B, and antibodies prevent the attachment of and infection by wild-type NiV. The EBOV GP is irrelevant in the context of a NiV vaccine. Previous studies demonstrated that a single intramuscular (IM) dose of rVSV expressing the NiV (Malaysia genotype) attachment glycoprotein (G) is highly attenuated and protected AGMs against lethal intratracheal challenge with NiV (Malaysia) virus (20). Our rVSV vaccine candidate was modified to express the NiV (Bangladesh) genotype, plaque-purified, manufactured to quality specifications, and studied in AGMs challenged with the more virulent (21) Bangladesh virus strain. This vaccine, code named PHV02, is now in clinical development (NCT05178901).

## Results

### Nipah virus challenge in control AGMs

All animals that received rVSV-EBOV succumbed to intranasal/intratracheal NiV challenge or met euthanasia criteria by day 7 to day 8 and had active NiV infections, viremia, clinical illness, and hematological and biochemical abnormalities. NiV viremia and shedding in oral swabs was evident by day 3 or 7 after challenge at titers of 3–6 log10 copies/mL. Lung radiograph abnormalities were seen by day 3, and radiograph scores increased by day 7. All AGMs in the control vaccine group developed typical signs of NiV disease in the last several days before death, including muddy or cyanotic mucous membranes, decreasing blood pressure, elevated respiratory rate, dehydration and/or vibrations felt over the chest wall, tachypnea, tachycardia, hypoxemia, and bloody/crusty nasal discharge. On day 7, most animals had increases in neutrophil and monocyte counts, mild thrombocytopenia, and increased hemoglobin and hematocrit. Increased serum transminases, creatinine and blood urea nitrogen, serum electrolyte abnormalities, and decreased total protein and albumin were observed variably. The lung pathology in the VSV-EBOV-vaccinated control animals (necropsied at day 7 to 8 after NiV-B infection) showed interstitial pneumonia with pulmonary edema, alveolar fibrin accumulation, leukocyte exudate within alveolar spaces, and epithelial or endothelial cell syncytia formation. Splenic lymphocytolysis was a common feature. At euthanasia, all rVSV-EBOV-vaccinated control animals displayed high genome copies (4–9 log10 copies/g) and infectious titers (4–5 log10 pfu/g) in respiratory and other selected tissues at euthanasia characteristic of a fulminant NiV-B infection (**Supplementary Figure S5**).

Only one of six AGMs in the group vaccinated on day 7 before challenge had detectable viremia and oral and nasal swabs following NiV challenge (low copy numbers 1–2 log 10 copies/mL). No other vaccinated animals had detectable viremia or shedding.

### Survival in vaccinated AGMs is dose-related and associated with an “all-or-nothing” immune response

**Figure 1:**
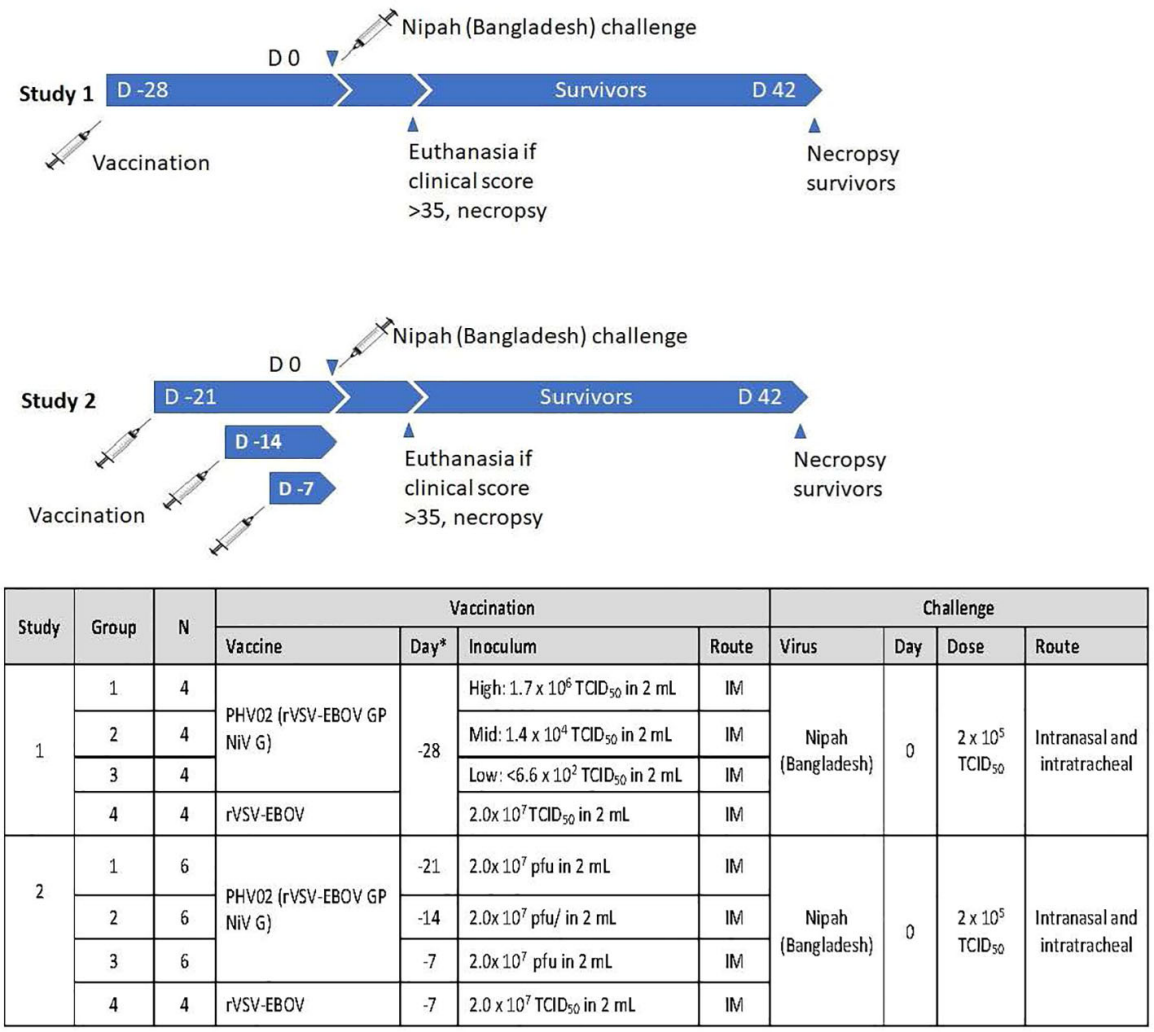
Experimental design of two studies in African green monkeys (AGMs) designed to test the protective efficacy of PHV02 and to define the immunological correlates of protection. D, Day.

**Figure 2:**
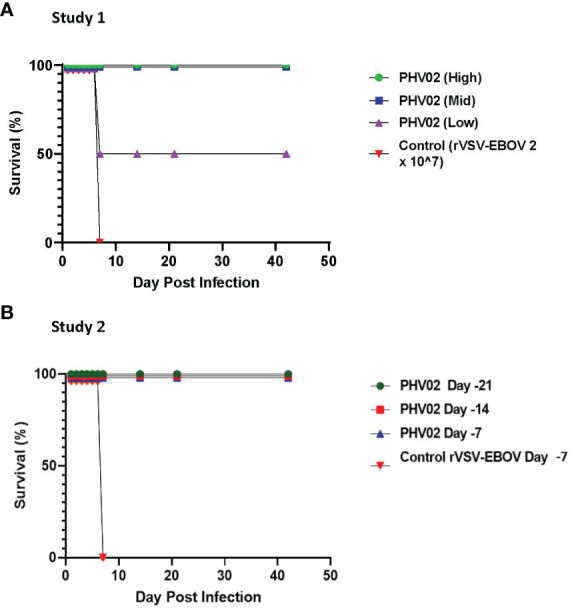
**(A)** Survival ratios, African green monkeys (AGMs) (N= 4 per group) vaccinated with graded doses of PHV02 vaccine 28 days before intranasal/intratracheal (IN/IT) challenge with 2 x 10^5^ TCID50 of Nipah (Bangladesh). Controls (n=4) received rVSV-EBOV. **(B)** Survival ratios, AGMs (N= 6 per group) vaccinated with PHV02 2 x 10^7^ pfu 21, 14 or 7 days before IN/IT challenge with 1 x 10^5^ TCID50 of Nipah (Bangladesh). Controls (n=2) received rVSV-EBOV 14 or 7 days before challenge.

In a first study (**Figure 1**), groups of four adult (>3 kg) male and female AGMs were given a single 2-mL IM (1 mL/caudal thigh) inoculation of graded doses of PHV02 (high: 1.7 × 10^6^, mid: 1.8 × 10^4^, or low: <6.6 × 10^2^) 50% tissue culture infectious doses (TCID_50_) based on back-titration of the diluted virus used for inoculation. The low dose is unknown and is based on the sensitivity (lower limit of quantitation, LLOQ) of the assay since no virus was observed in the back-titration of the material used for vaccination (see “Materials and methods”). The controls received rVSV-EBOV (2 × 10^7^ TCID_50_/mL in 2 mL). The AGMs were challenged on day 28 post-vaccination with approximately 1,000 LD_50_ (2 × 10^5^ TCID_50_) of NiV (Bangladesh) by the combined intranasal and intratracheal (IN/IT) routes. In total, 100% of the AGMs in the high and mid PHV02 dose groups survived and had no clinical signs, with no detectable viremia after challenge, whereas two animals in the low dose group (50%) and all four rVSV-EBOV control animals died or were euthanized because of severe clinical illness by day 7 (**Figure 2A**). Survival in the high and mid dose groups was significantly higher than in the rVSV-EBOV controls (*p* = 0.0047, log rank test), but there was no statistical difference between the PHV02 low dose and control group (*p* = 0.1573). All surviving animals were also protected against the clinical signs of illness and had low clinical scores and thoracic radiograph scores (**Supplementary Tables S1**–**S3**).

A second study was aimed at determining the time to onset of protective immunity. In this study (**Figure 1**), groups of six AGMs were given a single IM inoculation of 2 × 10^7^ pfu (the highest dose used in the phase 1 clinical trial) of PHV02 at 21, 14, or 7 days before IN/IT challenge with NiV (Bangladesh). The control animals (*n* = 4) were inoculated with rVSV-EBOV at 7 days before challenge. Day “0” was designated as the day of IN/IT challenge with NiV. All AGMs vaccinated with PHV02 on day -28 survived, whereas the control animals succumbed on day 8 after challenge (**Figure 2B**). The AGMs vaccinated on day -21 before challenge were also protected against viremia, clinical signs, and radiographic changes (**Supplementary Tables S2**–**S4**). All AGMs vaccinated on day -14 before challenge were protected against viremia, and only one of six animals vaccinated on day -7 before challenge had low-level viremia. Some animals vaccinated with PHV02 on day -14 or -7 had signs of clinical illness, but not as severe as in the controls, and all recovered).

**Figure 3:**
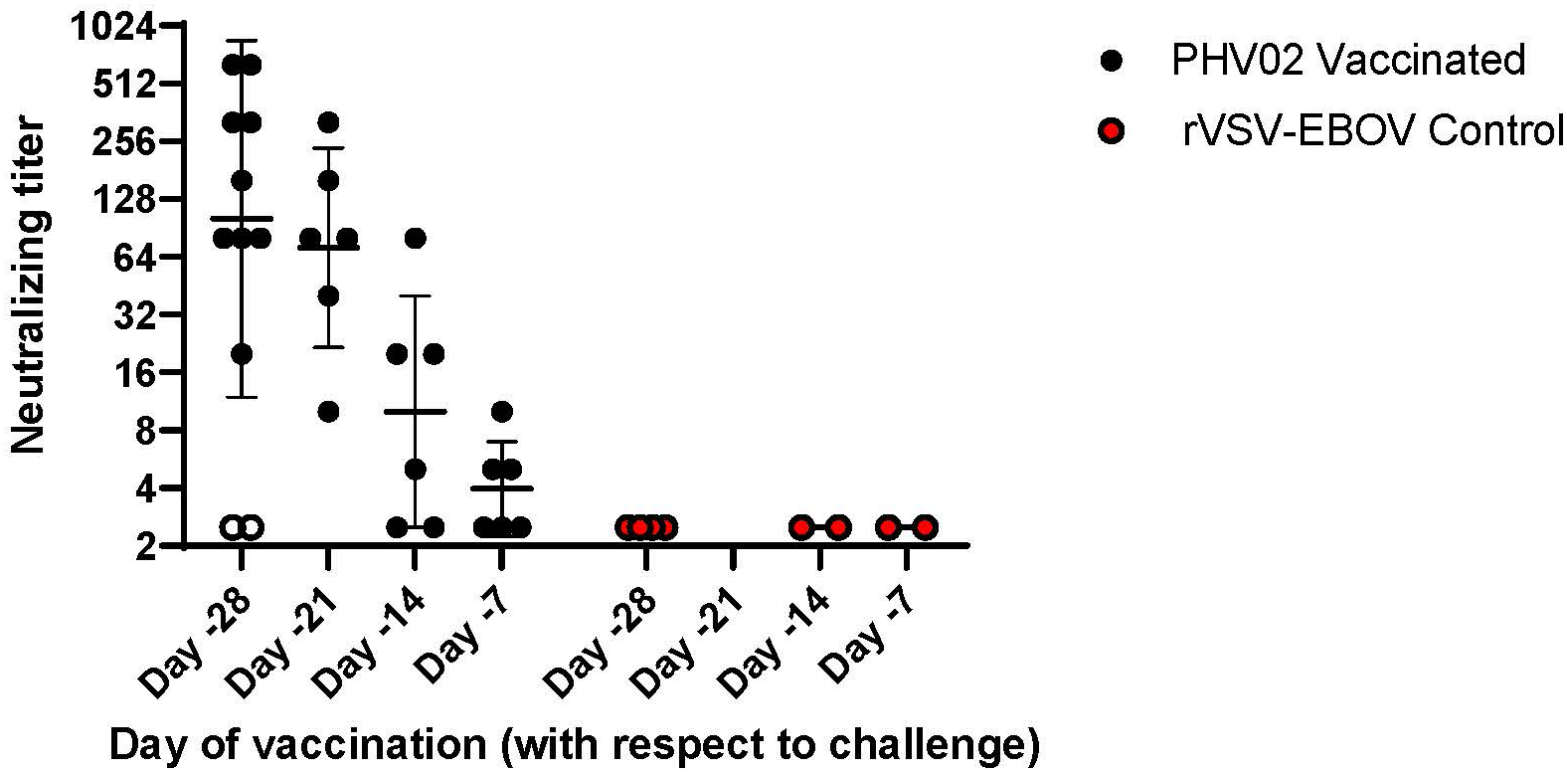
Pre-challenge (Day -1) Nipah virus neutralizing antibody titers, AGMs vaccinated with graded doses of PHV02 or with rVSV-EBOV (Controls) 28, 21, 14, or 7 days before IN/IT challenge (Day +1) with 2 x 10^5^ TCID50 of Nipah (Bangladesh). Animals in all dose groups in Study 1 are combined since there were no statistical differences between groups, (see text and **Supplemental Figure 1**). Day -1 neutralizing antibody titers are displayed for animals in study 2 that were vaccinated 21, 14 or 7 days before challenge. Individual animal titers and geometric mean (horizontal bar) and geometric mean standard deviation (GSD) are shown. The two animals in study 1 vaccinated with the low dose on day -28 that did not seroconvert and died are shown in open circles (o).

**Figure 4:**
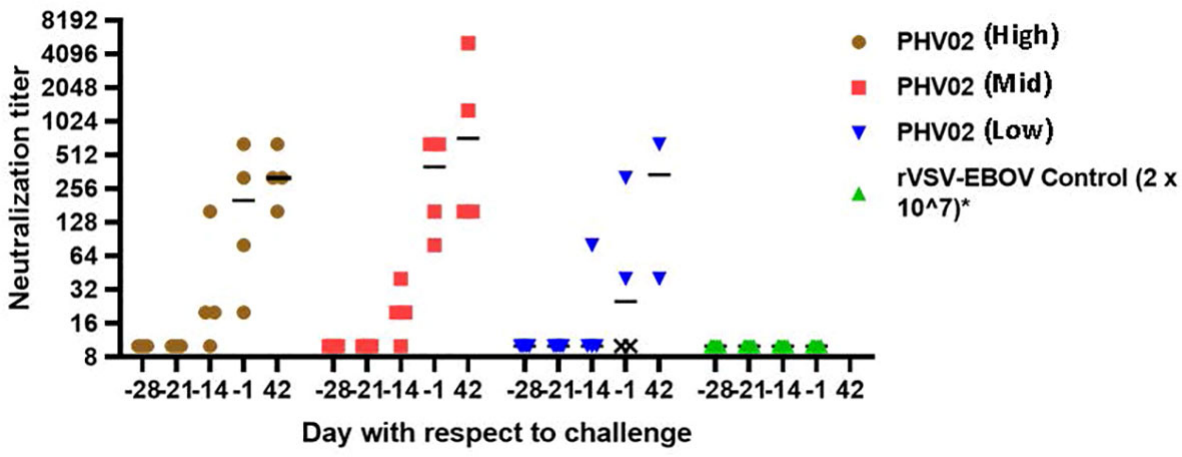
Nipah virus neutralizing antibody titers, AGMs vaccinated with graded doses of PH VO2 or with rVSV-EBOV(Controls) 28 days before IN/IT challenge (Day +1) with 2 x 10^5^ TCID50 of Nipah (Bangladesh), Study 1. Individual animal titers and geometric mean (horizontal bar) are shown by day with respect to challenge. The two monkeys in the low dose group that failed to develop neutralizing antibodies (designated X) died 7 days after challenge.

When vaccination was given 28 days (study 1) or 21 days (study 2) before challenge, all survivors mounted a neutralizing antibody response before challenge (**Figure 3**; **Supplementary Tables S1**, **S4**). In contrast, the two AGMs in study 1 in the PHV02 low dose group that succumbed to infection (**Figure 2A**) failed to develop detectable neutralizing antibodies before challenge (**Figure 4**—shown as “X” and **Figure 3**—shown as open circles; **Supplementary Tables S1**, **S4**). Excluding from analysis the two non-responders (low dose group) in study 1, there were no differences in geometric mean neutralizing antibody titers between the dose groups (two-way ANOVA for dose effect *p* = 0.4645), as shown also by overlapping 95% confidence intervals in **Supplementary Figure S1**. It was concluded that the effect of lowering dose was principally on seroresponse rather than antibody kinetics or titer, i.e., at very low dose levels, animals either responded or failed to respond, and among those that responded, dose level had no effect on antibody titer or kinetics of the response. Thus, down-dosing led to primary vaccine failure in two of four animals but did not elicit a sub-protective level of antibody.

### Protection is associated with low levels of neutralizing antibody

In study 2, vaccination performed 7 or 14 days before challenge afforded the opportunity to determine protection during the early phase of the adaptive immune response. The neutralization test was modified to test serial twofold dilutions of serum starting at 1:2.5 (serum dilution when mixed with virus = 1:5), allowing detection at a low (≥1:5) concentration of antibody and a more precise correlation with clinical and virological parameters.

#### Survival

In study 2, all monkeys vaccinated with PHV02 at 21, 14, or 7 days before challenge survived, whereas the rVSV-EBOV control animals vaccinated at 7 days before challenge succumbed on day 8 after challenge (**Figure 1B**).

All six AGMs in study 2 vaccinated on day -21 developed neutralizing antibodies pre-challenge (day -1), with a geometric mean titer (GMT) [ ± geometric mean standard deviation (GSD)] of 71.3 ( ± 3.3) (**Figure 3**; **Supplementary Table S4**). In the group vaccinated on day -14, four (67%) of six AGMs had a pre-challenge titer of ≥1:5 [GMT ( ± GSD) 10 ( ± 4.0)] (**Figure 3**; **Supplementary Table S4**). In the group vaccinated on day -7, two (33%) of six AGMs had detectable antibody pre-challenge [GMT ( ± GSD) 4 ( ± 1.8)]. It is possible that animals with no detectable antibody would be positive if sera were tested without dilution. Minimal protection, if any, was afforded by non-specific (innate) immunity since all control animals given the rVSV-EBOV vector on day -7 died or had similar survival times (day +8); the survival time was similar or up to 24 h longer than in study 1 where vaccination was 28 days before challenge.

Logistic regression analysis was performed to assess the relationship between pre-challenge (day -1) neutralizing antibodies titers and survival in studies 1 and 2 combined. For regression analysis, vaccinated AGMs with neutralizing titers below the lower level of quantitation (LLOQ) were assigned a value of LLOQ/2 (1:2.5), while control AGMs were arbitrarily assigned a value of 1.25 (LLOQ/4).

**Figure 5:**
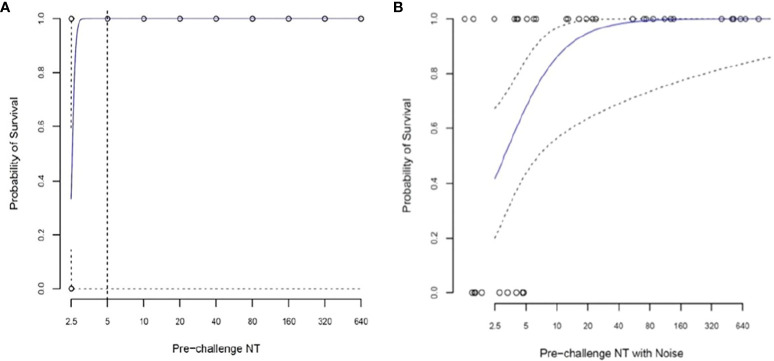
Logistic regression analysis to assess the relationship between pre-challenge (Day -1) log2 neutralizing antibody titer (NT) and survival in 38 African green monkeys (Studies 1 and 2 combined). **(A)** Simple logistic regression model with the sole explanatory variable pre-challenge log2 transformed NT. For a pre-challenge titer of 1:5, the predicted survival probability is 100%, but the confidence interval cannot be estimated. **(B)** Random noise (±1.0 log2) added to assess the impact of measurement error on predicted survival. For a pre-challenge titer of 1:5, the predicted survival probability (95% CI) is 73.2% (47.7, 89.1%).

A simple logistic regression model was used, in which the sole explanatory variable was pre-challenge log2-transformed neutralizing antibody titers. For a pre-challenge titer of 1:5, the predicted survival probability is 100%, but the 95% CI cannot be estimated (**Figure 5A**).

A second regression analysis was performed in which random noise (± 1.0 log2) was added to the pre-challenge antibody titers to assess the impact of measurement error on predicted survival. **Figure 5B** shows the predicted survival curve and its 95% CI. For a pre-challenge titer of 1:5, the predicted survival probability (95% CI) is 88.2% (45.2%, 100%).

The data indicate that any detectable neutralizing response before exposure to NiV was predictive of survival, but the absence of a response when vaccination occurs shortly (e.g., 7–14 days) before exposure does not predict lack of survival. As pointed out below, the survivors without pre-challenge antibody rapidly developed antibodies following challenge. The two AGMs in the low dose group that failed to mount an antibody response in study 1 had been vaccinated 28 days before challenge and represent true vaccine failures.

#### Clinical scores

**Figure 6:**
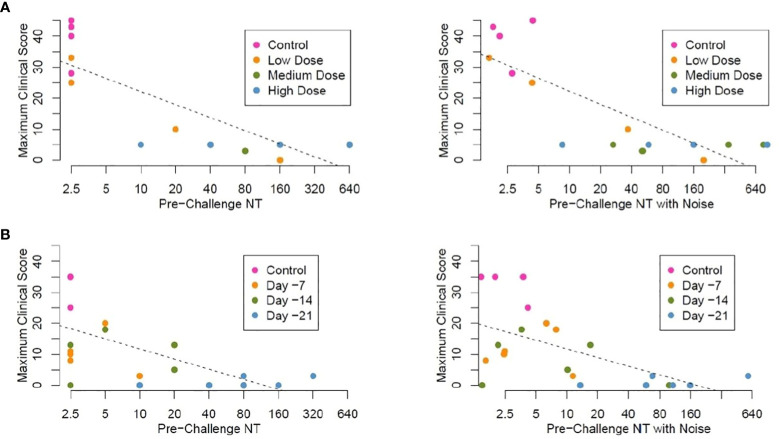
Linear regression analysis, maximum clinical scores by pre-challenge (Day -2) neutralizing antibody titers (NT) **(A)**. Study 1 Maximum clinical score by neutralizing titer pre-challenge (Day -1), animals vaccinated IM with graded doses of PHV02 28 days before IN/IT challenge with Nipah (Bangladesh). Regression analysis without (left panel) and with random noise [± 1.0log2) added to the pre-challenge NTs to assess the impact of measurement error] (right panel) **(B)**. Study 2 Maximum clinical score by neutralizing titer pre-challenge (Day -1), animals vaccinated IM with PHV02 (2x10^7^ pfu) 21, 14 or 7 days before or with rVSV-EBOV 14 or 7 days before IN/IT challenge with Nipah (Bangladesh) without (left panel) and with random noise (right panel). Individual data points are color coded by days from vaccination at the time of challenge. Note, in these plots in which noise has been added to pre-challenge titers unmask data points which are identical for different AGMs. For example, 3 control AGMs in Study 2 had the same maximum clinical score of 35. The estimated linear regression lines are superimposed on each plot.

Clinical scores were determined to assess whether AGMs without detectable antibody or with low pre-challenge titers in study 2 had evidence for active sub-lethal infections. All animals were scored daily by study staff experienced in signs of illness in the AGM model using a semi-quantitative grading scale (see “Materials and methods”). All rVSV-EBOV control animals had high clinical scores after NiV challenge (**Figure 6**; **Supplementary Table S4**). The AGMs vaccinated on day -21 were protected against illness and had pre-challenge (day -1) neutralizing antibodies (GMT 71, range 10–320). In contrast, four (67%) of six survivor AGMs vaccinated on day -14 before challenge and five (83%) of six survivor AGMs vaccinated on day -7 before challenge had signs of illness as indicated by moderate peak clinical scores. However, these clinical scores were significantly lower than those of the rVSV-EBOV controls (**Supplementary Figure S2**). The nonhuman primates manifesting illness either had no pre-challenge antibodies or had low neutralization titers (1:5-1:20). The mean clinical scores increased as the time between vaccination and challenge was reduced, but the differences were not statistically different as shown by overlapping 95% CI values (**Supplementary Figure S2**).

We performed a linear regression analysis of the maximum clinical scores in both studies as they relate to pre-challenge neutralizing antibody titer. The control AGMs were arbitrarily assigned a neutralizing titer of 1.25, while the vaccinated AGMs with titers below the LLOQ were assigned a neutralizing titer of 2.5. For the combined studies, the Pearson correlation coefficient was equal to −0.74 for the association between log_2_-transformed pre-challenge neutralizing titers and the maximum clinical score, indicating a moderate inverse correlation. **Figure 6A** plots pre-challenge titers without and with noise by maximum clinical score for study 1. **Figure 6B** plots pre-challenge titers without and with noise by maximum clinical score for the time-to-protection study 2. Based on these analyses, a pre-challenge titer of ≥1:40 was predictive of protection against the clinical signs of illness, i.e., maximum clinical scores 0–5.

#### Thoracic radiographic scores

Thoracic radiographs were taken at scheduled intervals after challenge (**Supplementary Table S3**), including at the time of euthanasia/death. Pulmonary changes progressed rapidly. In study 2, no or minimal infiltrates were present 1 day before euthanasia, and only two of four animals had radiographic evidence of pulmonary infiltrates at the time of euthanasia.

Regression analyses were performed for the association between log2-transformed day -1 pre-challenge neutralizing antibody titers and maximum thoracic radiographic score. For the combined studies, the Pearson correlation coefficient is equal to -0.51. In study 1, pre-challenge titers explain 67% of the variability in maximum thoracic radiograph score (*p* < 0.001). However, in study 2, pre-challenge titers only explain 13% of the variability (*p* = 0.087), principally because two of the rVSV-EBOV control monkeys which had severe clinical scores requiring euthanasia had not developed evidence of significant pulmonary infiltrates (**Supplementary Tables S3**, **S4**).

**Supplementary Figure S3A** plots the pre-challenge titers without and with noise by maximum thoracic radiograph score for study 1. Individual data points are color-coded by dose of vaccine received. **Supplementary Figure S3B** plots the pre-challenge titers without and with noise by maximum thoracic radiograph score for study 2.

In study 1, a pre-challenge titer of ≥20 was predictive of protection against pulmonary infiltrates caused by NiV challenge infection. In study 2, two (50%) of four AGMs in the control group did not develop pulmonary infiltrates, making the correlation with antibody level less clear.

#### Post-challenge antibody

In AGMs in study 1 and study 2, a pre-challenge neutralizing titer of ≥1:40 predicted the absence of a post-challenge an approximately fourfold rise in titer in the majority of the AGMs (**Supplementary Figure S4**). There were only two (7%) of 28 AGMs [one in study 1 (vaccinated on day -28 before challenge) and one in study 2 (vaccinated on day -21 before challenge)] with pre-challenge (day -1) titers ≥1:40 that had an antibody rise after challenge. A titer of ≥1:40 was thus sufficient to an anamnestic immune response in most vaccinated AGMs. The lack of an antibody response following virus challenge may be ascribed to the neutralization of incoming virus, preventing replication (“sterilizing immunity”). The AGMs in the days -14 and -7 treatment groups had low pre-challenge titers (**Supplementary Table 4**), and the antibody response following challenge is indicative of a response to both the infection and vaccination since antibodies elicited by the latter are still evolving. However, a neutralizing titer of ≥1:40 before a severe respiratory NiV challenge appeared to abrogate virus replication and prevent an antibody response. The probability of having an approximately fourfold rise in antibody was significantly reduced at a pre-challenge titer of ≥1:40 (*p* = 0.0001, Fisher’s exact test, two-sided).

#### Tissue RNA levels at necropsy

Necropsies were performed on vaccinated survivors on day +41 or +42 after challenge on day 0 and on low dose PHV02 non-survivors and rVSV-controls at time of death/euthanasia (7 or 8 days after challenge). In study 1, all controls and the two non-survivors in the PHV02 low dose group had high levels of RNA (5–10 log_10_ copies/g) and infectious virus (3–6 log_10_ TCID_50_) in multiple tissues including the lung, spleen, and brain on necropsy on day +7 (data not shown). In contrast, the necropsy of survivors on day +41 revealed only one animal (NiV 117 in the low dose group, **Supplementary Table 1**) with low levels of detectable genomic RNA and no detectable infectious virus in any tissue (RNA was found in the right middle and lower lobes of the lung, 2.7–3.3 log_10_ copies/g). Interestingly, this AGM with a low viral load in the lung had a low level of antibody (1:40) both pre-challenge and on day 41 and did not seroconvert after challenge, possibly indicating less robust viral clearance.

In study 2, all rVSV-EBOV control animals had high levels of genomic RNA (4.8–9.5 log_10_ copies/g) in multiple tissues on necropsy on day 8 after challenge, especially in lung segments and conducting portions of the lower respiratory tract, brain, spleen, and urinary bladder. In vaccinated AGMs, substantially lower levels of detectable genomic RNA (3–4 log_10_ copies/g) were present in one or more tissue samples, with no differences across the day -21, -14, and -7 vaccine groups (**Supplementary Figure S5**).

### Protection by vaccination shortly before challenge may depend on the post-challenge immune response

**Figure 7:**
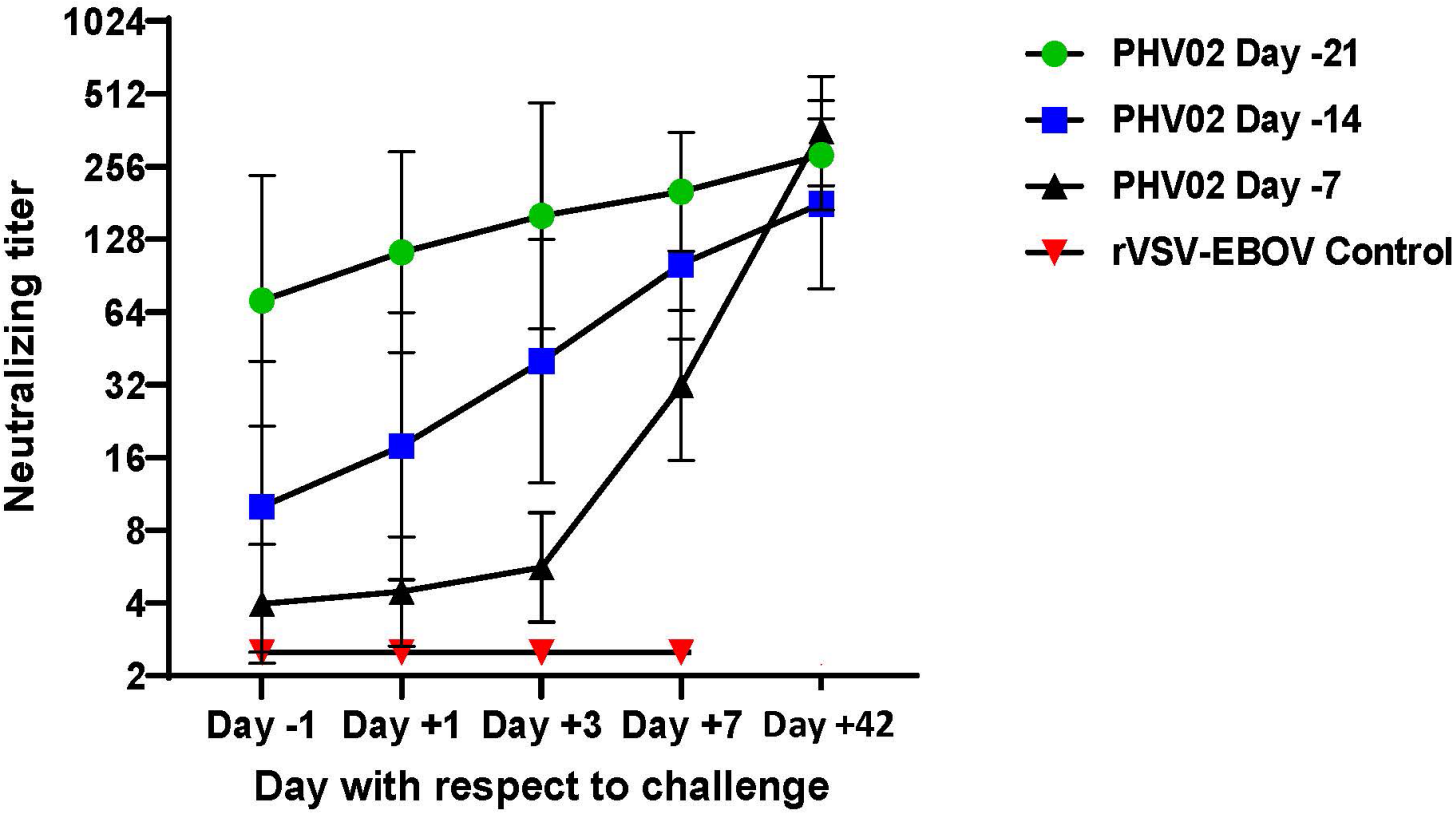
Geometric mean (±GSD) Nipah virus neutralizing antibody titers following challenge (Day +1) with 2 x 10^5^ TCID50 of Nipah (Bangladesh). AGMs were vaccinated with graded doses of PHV02 or with rVSV-EBOV (Controls) 21, 14, or 7 days before IN/IT challenge.

As described above, the AGMs in study 2 vaccinated at a short interval (days -14 or -7) before NiV challenge had no detectable (<1:5) or had low titers (1:5–20) of neutralizing antibodies on day -1 before challenge. To determine whether the immune response during the challenge virus incubation period abrogated the infection and resulted in survival, we determined neutralizing titers on days +1, +3, and +7 days after challenge. All AGMs in both the day -14 and -7 treatment groups developed neutralizing antibodies or showed an increase in antibody titer during the 7 days following challenge, whereas the rVSV-EBOV control animals that died on day 8 had no detectable response to Nipah challenge (**Figure 7**). The largest increase in GMT was seen in the animals vaccinated on day -7 before challenge. The post-challenge response in the day -14 and day -7 groups reflects both the evolution of the response to vaccination and the booster effect of challenge, the latter being most evident in the day -7 vaccine group. The response attributable to the vaccine alone can be estimated from the antibody kinetics without challenge when compared to the observed response after challenge (**Supplementary Figure S6**). While the post-challenge response was greater than the response expected for the same intervals following vaccination without challenge, the differences were not statistically significant.

These results indicated that vaccination given within 7–14 days of a severe virus challenge protected against death and that survival was associated with the appearance of low levels of neutralizing antibodies before infection or shortly after infection, which increased during the incubation period of the challenge infection.

## Discussion

In order to assess the relationship between immune response and protection and the potential translation to humans, it is necessary to consider the severity and time course of infection in the challenge model. The AGM is widely accepted as the preferred nonhuman primate model of NiV disease since other species, in particular macaques, do not develop consistently fatal illness (22). The course of NiV infection in the AGM model is more active and lethal than in humans. The 100% mortality ratio in the AGM model is higher than reported in humans (in recent outbreaks caused by the Bangladesh genotype, 73%–89%) (23). The IN/IT route of challenge and high challenge dose (1,000 LD50) likely promote a rapidly progressive pulmonary infection. Survival time in AGMs (7 to 8 days) is shorter than in humans, in which the median incubation period is approximately 10 days and the average duration of hospital stay is 1–9 days (24, 25). The IN/IT route in AGMs may be a model of respiratory exposure and inter-human transmission (26), although human NiV infections also occur following oral ingestion of contaminated date palm sap and probably other modes of contact spread. The outcome of infection and pathogenesis with respect to the onset of pulmonary and neurological infection is dependent on the challenge dose and route (27, 28),. It may be concluded that the AGM model, high challenge dose, and IT route of infection provide a severe test of immunity afforded by a vaccine. If human immune responses can be shown to be similar to those associated with protection in this lethal model, they would likely indicate that the vaccine would provide clinical benefit.

A limitation of the studies reported here is that only humoral immunity (IgG binding and neutralizing antibodies) was investigated, whereas T cells undoubtedly contribute to protective immunity and recovery. Viral clearance mediated by cytotoxic T cells may be necessary to prevent persistent and recurring NiV virus infections (9). In a previous study of the rVSV vaccine expressing NiV G (Malaysia), Prescott et al. found significantly increased CD8+ T cells expressing granzyme B or interferon-γ in vaccinated vs. control animals at 3 and 7 days post-challenge, respectively (20). Prasad and colleagues concluded that both antibodies and T cells contributed to survival in cynomolgus macaques following NiV challenge (22). Antibodies play a major role in pre-exposure protection as shown by passive transfer studies (10, 11), and neutralization is believed to be the principal functional activity (8). Other Fc-mediated functions of antibody, such as cellular phagocytosis, were also not measured in our studies and could potentially contribute to protection. We did, however, measure IgG-binding antibodies by ELISA; these analyses did not suggest that non-neutralizing antibodies were a better marker of protection than neutralization. Indeed IgG ELISA and neutralizing titers were closely correlated (**Supplementary Figure S7**), and low levels of neutralizing antibodies were detected without a detectable binding antibody, which is likely due to the lower dilution of serum in the neutralization test (1:5) than in ELISA (1:100).

In the first (dose–response) study, PHV02 vaccine was administered 28 days before NiV challenge. The lowest pre-challenge neutralizing titer was 1:20, and that animal was fully protected against illness, death, and residual viral RNA in tissues collected at necropsy 42 days after challenge. There were two animals in the lowest dose group (<6.6 × 10^2^ pfu) that did not seroconvert and developed fatal illness with survival time equivalent to the control animals. The results indicated a very low dose of vaccine which may result in a protective response or in primary vaccine failure. This “all-or-nothing” response is not surprising for a replication-competent viral vaccine that expands its antigenic mass after inoculation. In the case of the replication-competent rVSV-EBOV vaccine, reduction in dose by 10-fold increments to low levels resulted in increasing rates of primary vaccine failure (29).

In the second study, AGMs were challenged 21, 14, or 7 days after vaccination, during the early phase of the adaptive immune response, with the objective of determining a level of antibody predictive of survival. All animals vaccinated at those intervals before challenge were protected against death. All AGMs survived whether or not they had detectable neutralizing antibodies the day before challenge. In those animals with pre-challenge titers <1:5, antibodies appeared rapidly after challenge (**Figure 7**) and likely abrogated the infection during the first few days after challenge. Nine (75%) of 12 AGMs vaccinated on day -14 or -7 developed signs of illness, from which they recovered, and the clinical scores were substantially lower than in the control animals (**Figure 6B**; **Supplementary Table S4**). Of the three AGMs that developed no signs of illness, two had detectable neutralizing antibodies before challenge.

Overall, based on results in a severe challenge model, it may be concluded that any detectable antibody level is predictive of survival against challenge performed up to 28 days after vaccination, that vaccination within as few as 7 days of exposure may protect against severe illness and death, and that the immediate post-challenge immune response protects even where pre-challenge antibodies are low or undetectable. The results are consistent with the observation in a phase 3 clinical trial of the rVSV-EBOV vaccine, in which no one who had been vaccinated within the previous 10 days developed Ebola virus disease (30).

rVSV vectors are known to activate multiple antiviral genes (31). To control for innate immunity in our studies, the animals were given rVSV-EBOV concurrent with PHV02 28 days (study 1) or 7 and 14 days (study 2) before challenge. AGMs given rVSV-EBOV 7 days before challenge had a similar survival time as those challenged at longer intervals, suggesting that, at an interval of ≥7 days, residual innate immunity did not provide protection. None of the control AGMs mounted a neutralizing antibody response to the NiV challenge before death. In contrast to our study, a rVSV vaccine against Marburg virus (MARV) disease given 7 or even 3 days before MARV challenge was shown to protect nonhuman primates; in that experiment, the day -3 survivors were characterized by strong antiviral gene activation, and protection was attributed to innate rather than adaptive immunity (32). Peri-exposure vaccination of nonhuman primates with rVSV-EBOV given 1 or 24 h after challenge protected the animals against both EBOV and the heterologous MARV, again indicating a role for innate immunity in early protection (33). The same conclusion was reached in the case of peri-exposure vaccination of hamsters with the rVSV-Nipah vaccine (34). In our study, we did not evaluate protection by vaccination given after challenge or as early at 3 days before challenge. However, the protection seen in animals vaccinated with PHV02 7 days or more before challenge was attributable to the adaptive immune response which was detected either before or shortly after challenge. Although T cells were not assessed and may have contributed to protection, the neutralizing antibody response provided an adequate explanation for pre-exposure protection.

In our studies, neutralizing antibody induced by PHV02 at ≥1:5 was established as a marker of survival. More work will be required to establish a quantitative level of protection, although the data presented here suggest that a titer of ≥1:40 before exposure was associated with the absence of clinical signs (**Figure 6**) and protected against an increase in antibody levels post-challenge, which is indicative of sterilizing immunity (**Supplementary Figure S3**). Using a different model system (Balb/c mice immunized with an HIV pseudovirus expressing NiV G protein and challenge followed by *in vivo* bioluminescence imaging), Nie et al. found a neutralizing antibody level of 170 or greater to be protective (16).

NiV challenge in control AGMs caused a rapidly fatal illness and necropsy tissues, including lung segments and brain that had high levels of viral RNA. In AGMs vaccinated with PHV02 21 or 28 days before challenge, solid immunity was established and protected against death, clinical illness, and infection of vital organs. Nearly all AGMs vaccinated 28 days before challenge had cleared viral RNA by the time of necropsy on day +42. In animals vaccinated at shorter intervals (days -21, -14, or -7) before challenge, the immune response was sufficient to prevent death, but there was residual RNA at low levels in the lung and brain at day 42 (**Supplementary Figure S5**). This finding indicated that the vaccine given shortly before exposure had abrogated but not prevented infection and that the abortive infection with NiV had resulted in neuroinvasion. Since humans surviving a natural infection with NiV can occasionally develop late-onset or relapsing encephalitis (7) it cannot be excluded that persons vaccinated and subsequently exposed to NiV could be protected against the acute disease but have persistent infection of immune-privileged sites in the brain. Liu et al. also reported subclinical encephalitis with persisting genomic RNA in neurons and glial cells in the brains of nonhuman primates that had survived the acute phase of NiV disease following IT virus challenge (35). Based on the AGM model, it would appear that this phenomenon is more likely to occur in the setting of vaccination shortly before exposure during the development of the early immune response.

In these studies, vaccination with PHV02 predicts a positive outcome—survival and abrogation of disease with neutralizing antibody seroresponse as a putative biomarker of protection. PHV02 vaccine is a promising candidate for the development of a human vaccine against Nipah disease.

### Ethical statement

The use of study animals was approved by the Institutional Animal Care and Use Committee (IACUC) of the Rocky Mountain Laboratories, and the experiments were performed in an AAALAC International-accredited facility following institutional guidelines for animal use, the guidelines and basic principles in the NIH Guide for the Care and Use of Laboratory Animals, the Animal Welfare Act, USDA, and the USPHS Policy on Humane Care and Use of Laboratory Animals. Humane endpoint criteria, specified and approved by the IACUC, were applied to determine when animals should be humanely euthanized.

## Materials and methods

### Vaccines and challenge virus

PHV02 (rVSVΔG-EBOV GP-NiV G) is a plaque-purified rVSV (Indiana) virus with the VSV envelope glycoprotein (G) deleted and replaced with the genes for the surface glycoprotein (GP) of Ebola Zaire (EBOV), which mediates membrane fusion between the virus and the host cell, and the NiV G protein) which binds to ephrin-B2 and ephrin-B3 cell receptors and against which NiV-protective antibodies are raised. The control in animal studies is rVSVΔG-EBOV GP [rVSV-EBOV (Kikwit 1995)], similar to the approved Ebola vaccine. rVSV-EBOV is a similar construct to PHV02 but lacks the expression of NiV G protein. Both viruses are replication competent and highly attenuated in various animal models compared to the parental viruses (i.e., VSV, EBOV, NiV) (36). Both vaccine viruses were produced in Vero cell cultures. The NiV challenge virus is a human isolate, Nipah (Bangladesh, 200401066, GenBank AY988601), passaged three times in Vero cells and frozen in Dulbecco’s Minimum Essential Medium (DMEM)–10% fetal bovine serum.

The doses used in the studies deserve further description. In study 1, AGMs were inoculated with a range of doses targeting 2 × 10^7^, 2 × 10^6^, and 2 × 10^5^ pfu (in 2 mL given IM) based on the release titer of the virus by the manufacturer using a qualified potency assay. The inocula were back-titrated by cytopathic effects assay in Vero cells at the facility where the animal study was performed (NIAID). The back-titration showed that virus doses were lower than expected (1.7 × 10^6^, 1.8 × 10^4^, or <6.6 × 10^2^ TCID_50_, which are the high, mid, and low doses described in the “Results” section, respectively. The low dose could not be determined since it is below the LLOQ of the assay. The virus content of the inocula on back-titration was confirmed by the manufacturer, which then conducted an investigation showing that the diluent (0.9% saline for injection) used by the NIAID to prepare the dosing materials have low pH (6-6.2), which resulted in a loss of infectivity. For study 2, the diluent used for preparation of the dosing material was changed to DMEM, which resulted in no loss of titer.

#### Animal procedures

Commercially available monkey chow, treats, and fruits/vegetables were provided twice daily. Water was available *ad libitum*. Environmental enrichment consisted of a variety of human interactions, manipulanda, movies, and music. The AGMs were acclimatized to the study room for a minimum of 5 days. The animals were identified by cage cards and tattoos numbers. The animals were randomly divided into treatment groups and allowed to acclimate to the study room for a minimum of 5 days. All study activities were conducted with the animals under ketamine or Telazol anesthesia. The animals were inoculated IM in the caudal thigh with study vaccines and held for varying intervals before NiV challenge. The monkeys were monitored at least twice daily for any adverse effects to vaccination or signs of disease upon challenge. At protocol-specified intervals, thoracic radiographs, blood, and swabs (nasal, oral, and in some animals, rectal) were collected for clinical laboratory, virology, and immunological tests. Blood was drawn from the femoral, saphenous, or cephalic vein on exam days using a 21–26-gauge needle. The exact volume of blood collected did not exceed 15% of the total circulating blood volume in any 2-week period.

#### Nipah challenge

Nonhuman primates were given a total dose of 2 × 10^5^ TCID50 NiV (Bangladesh) divided equally over two routes: intratracheal (IT; 4 mL) inoculation was accomplished under anesthesia using a feeding tube advanced down an orally placed endotracheal tube, and intranasal (IN; 0.5 mL into each nostril) inoculation was performed by dripping the inoculum into each nostril using a micropipette.

#### Clinical endpoints and euthanasia

Experienced staff observed the AGMs and recorded clinical signs using a semiquantitative scoring sheet. The AGMs in severe respiratory distress (open mouth breathing with lack of activity and cyanosis) or with evidence of bloody or purulent discharge from the respiratory tract or severe neurological signs [seizure activity, neurologic signs that interfere with the ability to ambulate or ingest food or water, unconsciousness or moribundity (no or little response to human presence and prompting)] or with body temperature <35°C were euthanized. Any AGM with a clinical score >35 was euthanized.

#### Thoracic radiographs

Ventro-dorsal and lateral thoracic radiographs were done while the AGMs are under anesthesia on specified examination days and at euthanasia/death. Radiographs were evaluated and scored for the presence of pulmonary infiltrates by two board-certified laboratory animal veterinarians according to a standard scoring system (37). Briefly, each lung lobe (upper left, middle left, lower left, upper right, middle right, lower right) was scored individually based on the following criteria: 0 = normal examination; 1 = mild interstitial pulmonary infiltrates; 2 = moderate interstitial pulmonary infiltrates, perhaps with partial cardiac border effacement and small areas of pulmonary consolidation (alveolar patterns and air bronchograms); and 3 = pulmonary consolidation.

Thoracic radiograph findings are reported as a single radiograph score for each AGM on each exam day. To obtain this score, the scores assigned to each of the six lung lobes were added together and recorded as the radiograph score for each animal on each exam day. Scores may range from 0 to 18 for each animal on each exam day.

#### Neutralization

Serial twofold dilutions of heat-inactivated test sera were made in DMEM with 2% heat-inactivated fetal bovine 1 mM L-glutamine, 50 U/mL penicillin, and 50 μg/mL streptomycin, mixed with 100 TCID_50_ of NiV (Bangladesh) virus, incubated (37° C, 60 min), and inoculated onto Vero E6 cells grown in monolayer cultures in 96-well plates. The cells were incubated for 5 to 6 days. Virus neutralization titer is the highest dilution of serum for which no cytopathic effects were observed.

#### IgG ELISA

Nunc MaxiSorp™ flat-bottom microplates were coated with gamma-irradiated NiV (Bangladesh) in phosphate-buffered saline overnight at 2–8°C. After the removal of coating, antigen plates were blocked 15 min at room temperature with 5% skim milk in 1X DPBS with 0.05% Tween 20. The plates were washed three times with 1x DPBS containing 0.5% Tween 20. Sera were added beginning at 1:100 through 1:6,400 using serial fourfold dilutions and incubated for 1 h at room temperature. The plates were washed three times with 1x DPBS containing 0.5% Tween 20, and secondary antibody (Southern Biotech 6200-005) was applied at 1:1,000 dilution in blocking buffer and incubated for 1 h at room temperature. After the removal of detecting antibody, the plates were washed six times with 1x DPBS containing 0.5% Tween 20. ABTS substrate was added and incubated for 15 min at room temperature and then stopped with 5% SDS in H_2_O. Absorbance at 405 nm was read within 30 min of stopping the reaction. ELISA titers were calculated by taking both the average and standard deviation of negative control results. The standard deviation ×3 + the average of the control wells set the negative upper threshold. Only samples with ODs greater than the standard deviation x3 above the average negative results were considered to be positive.

#### Quantitative PCR

RNA was extracted from swab samples in DMEM and from EDTA blood samples using the QIAamp viral RNA mini kit (Qiagen) according to the manufacturer’s instructions. RNA was extracted from tissues using the RNeasy kit (Qiagen); tissues (30 mg) were homogenized in RLT buffer (Qiagen), and RNA was extracted according to the manufacturer’s instructions. Moreover, 5 µL RNA was used in a one-step real-time RT-PCR targeting the N gene of NiV using the Rotor-Gene probe kit (Qiagen) according to the instructions of the manufacturer. In each run, dilutions of PCR standards with known copy numbers were run in parallel to calculate copy numbers in the samples. qRT-PCR assays and standards specific for NiV (Bangladesh) or VSV were used.

### Statistical methods

Regression analyses were performed with the open-source program R version 4.2.0 (https://www.R-project.org/). GMT, GSD, 95% CI, and group comparison analyses were determined using GraphPad Prism 9, with the test method specified in the text.

## Data availability statement

The original contributions presented in the study are included in the article/**Supplementary Material**. Further inquiries can be directed to the corresponding author.

## Ethics statement

The animal study was approved by NIAID Institutional Animal Care and Use Committee. The study was conducted in accordance with the local legislation and institutional requirements.

## Author contributions

TM authored the manuscript; TM and RN designed the studies; FF, AG, EH, JC, KM-W, AO, JL, PH, CC, and CS carried out the experimental procedures and virological, clinical, radiographic and pathological tests; WR performed statistical analyses; and JF provided guidance and served as principal investigator on the grant from CEPI that supported the work. All authors contributed to the article and approved the submitted version.
