# Supplementary material for: Immunological correlates of protection afforded by PHV02 live, attenuated recombinant vesicular stomatitis virus vector vaccine against Nipah virus disease
Source: Front Immunol. 2023 Sep 4;14:1216225. doi: 10.3389/fimmu.2023.1216225 (PMC10507387; doi:10.3389/fimmu.2023.1216225)
Supplement: Supplementary file 1 [file DataSheet_1.docx]

**Supplementary Information**

**Immunological Correlates of Protection Afforded by PHV02 live, attenuated recombinant vesicular stomatitis virus (rVSV) vector vaccine against Nipah Virus Disease**

Thomas P Monath, Richard Nichols, Friederike Feldmann, Amanda Griffin, Elaine Haddock, Julie Callison, Kimberly Meade-White , Atsushi Okumura, Jamie Lovaglio, Patrick W. Hanley, Chad Clancy, Greg Saturday, Wasima Rida, Joan Fusco

**Supplementary Table 1.** Study 1 AGMs vaccinated with PHV02 or rVSV-EBOV (control) 28 days before challenge: neutralizing antibody titers by day with respect to challenge, outcome, and post-challenge viremia, clinical and radiograph scores, and antibody response

| **Monkey** | **Treatment (Day -28)** | **Neutralizing antibody titer** | | | | | | **Nipah viremia Day +1, +3, +7** | **Peak challenge viremia (log10 copies/mL) [Day]** | **Maximum clinical score** | **Maximum lung radiograph score** | **≥4-fold antibody rise post challenge** |
| --- | --- | --- | --- | --- | --- | --- | --- | --- | --- | --- | --- | --- |
|  |  | **Day -28** | **Day -21** | **Day -14** | **Day -1** | **Day +42** | **Survived?** |  |  |  |  |  |
| NiV 123 | PHV02 High dose (1.7 x 10^6^ pfu) | <20 | <20 | **160** | **640** | **640** | Yes | No | ─ | 5 | 2 | No |
| NiV 124 |  | <20 | <20 | <20 | **20** | **160** | Yes | No | ─ | 5 | 2 | Yes |
| NiV 125 |  | <20 | <20 | **20** | **80** | **1280** | Yes | No | ─ | 5 | 2 | Yes |
| NiV 126 |  | <20 | <20 | **20** | **320** | **320** | Yes | No | ─ | 5 | 2 | No |
| NiV 119 | PHV02 Mid dose (1.8 x 10^4^ pfu) | <20 | <20 | **20** | **640** | **1280** | Yes | No | ─ | 5 | 2 | No |
| NiV 120 |  | <20 | <20 | **40** | **640** | **5,120** | Yes | No | ─ | 5 | 2 | Yes |
| NiV 121 |  | <20 | <20 | <20 | **80** | **160** | Yes | No | ─ | 5 | 2 | No |
| NiV 122 |  | <20 | <20 | **20** | **160** | **160** | Yes | No | ─ | 3 | 1 | No |
| NiV 115 | PHV02 Low dose (<6.6 x 10^2^ pfu) | <20 | <20 | <20 | <20 |  | No | Yes | 6.0 [+7] | 33.0 | 13 | ─ |
| NiV 116 |  | <20 | <20 | <20 | <20 |  | No | Yes | 4.3 [+7] | 25 | 13 | ─ |
| NiV 117 |  | <20 | <20 | <20 | **40** | **40** | Yes | No | ─ | 10 | 1 | No |
| NiV 118 |  | <20 | <20 | **80** | **320** | **640** | Yes | No | ─ | 0 | 3 | No |
| NiV 111 | rVSV-EBOV Control (2 x 10^7^ pfu) | <20 | <20 | <20 | <20 |  | No | No | ─ | 43 | 8 | ─ |
| NiV 112 |  | <20 | <20 | <20 | <20 |  | No | Yes | 5.7 [+7] | 40 | 7 | ─ |
| NiV 113 |  | <20 | <20 | <20 | <20 |  | No | Yes | 6.3 [+7] | 28 | 8 | ─ |
| NiV 114 |  | <20 | <20 | <20 | <20 |  | No | Yes | 6.8 [+7] | 45 | 12 | ─ |

**Supplementary Table 2.** Clinical scores by treatment group and day after Nipah (Bangladesh) IN/IT challenge on Day 0, Studies 1 and 2

1. **Study 1**

| **Monkey** | **Treatment** | **D0** | **D1** | **D2** | **D3** | **D4** | **D5** | **D6** | **D7** | **D8** | **D9** | **D10** | **D11** | **D12** | **D13** | **D14** | **D15** |
| --- | --- | --- | --- | --- | --- | --- | --- | --- | --- | --- | --- | --- | --- | --- | --- | --- | --- |
| NiV 123 | PHV02 High dose (1.7x 10^6^ pfu) | 0 | 0 | 5 | 0 | 0 | 0 | 0 | 0 | 5 | 0 | 0 | 0 | 0 | 0 | 0 | 0 |
| NiV 124 |  | 0 | 0 | 5 | 0 | 0 | 0 | 0 | 0 | 5 | 5 | 0 | 0 | 0 | 5 | 0 | 5 |
| NiV 125 |  | 0 | 0 | 5 | 0 | 5 | 5 | 0 | 5 | 5 | 5 | 0 | 5 | 0 | 0 | 0 | 0 |
| NiV 126 |  | 0 | 5 | 3 | 0 | 5 | 0 | 0 | 5 | 5 | 5 | 0 | 5 | 0 | 5 | 0 | 5 |
| NiV 119 | PHV02 Mid dose (1.8 x 10^4^ pfu) | 0 | 0 | 5 | 0 | 5 | 5 | 5 | 5 | 5 | 5 | 0 | 5 | 0 | 5 | 0 | 5 |
| NiV 120 |  | 0 | 5 | 5 | 5 | 3 | 3 | 3 | 3 | 0 | 5 | 0 | 5 | 5 | 0 | 5 | 5 |
| NiV 121 |  | 3 | 3 | 5 | 3 | 3 | 3 | 3 | 3 | 5 | 3 | 3 | 3 | 3 | 0 | 3 | 3 |
| NiV 122 |  | 3 | 3 | 3 | 3 | 3 | 3 | 3 | 3 | 3 | 3 | 3 | 0 | 0 | 3 | 0 | 3 |
| NiV 115 | PHV02 Low dose (<6.6 x 10^2^ pfu) | 0 | 5 | 10 | 15 | 23 | 20 | 20 | 33 |  | | | | | | | |
| NiV 116 |  | 0 | 0 | 3 | 3 | 8 | 8 | 8 | 25 |  | | | | | | | |
| NiV 117 |  | 0 | 0 | 3 | 10 | 3 | 3 | 3 | 5 | 5 | 0 | 0 | 3 | 3 | 3 | 0 | 0 |
| NiV 118 |  | 0 | 0 | 0 | 0 | 0 | 0 | 0 | 0 | 0 | 0 | 0 | 0 | 0 | 0 | 0 | 0 |
| NiV 111 | rVSV-EBOV Control (2 x 10^7^ pfu) | 0 | 5 | 3 | 8 | 8 | 13 | 23 | 43 |  | | | | | | | |
| NiV 112 |  | 0 | 0 | 5 | 10 | 10 | 13 | 26 | 40 |  | | | | | | | |
| NiV 113 |  | 0 | 3 | 0 | 0 | 0 | 10 | 28 | 28 |  | | | | | | | |
| NiV 114 |  | 0 | 5 | 5 | 10 | 10 | 20 | 25 | 45 |  | | | | | | | |

1. **Study 2**

| **Monkey** | **Treatment** | **D0** | **D1** | **D2** | **D3** | **D4** | **D5** | **D6** | **D7** | **D8** | **D9** | **D10** | **D11** | **D12** | **D13** | **D14** | **D15** |
| --- | --- | --- | --- | --- | --- | --- | --- | --- | --- | --- | --- | --- | --- | --- | --- | --- | --- |
| NIV 127 | PHV02 (2 x 10^7^ pfu) Day -21 | 0 | 0 | 0 | 0 | 0 | 0 | 0 | 0 | 0 | 0 | 0 | 0 | 0 | 0 | 0 | 0 |
| NIV 128 |  | 0 | 0 | 0 | 0 | 0 | 0 | 0 | 0 | 0 | 0 | 0 | 0 | 0 | 0 | 0 | 0 |
| NIV 129 |  | 0 | 0 | 0 | 0 | 0 | 0 | 3 | 0 | 0 | 0 | 0 | 0 | 0 | 0 | 0 | 0 |
| NIV 130 |  | 0 | 0 | 0 | 0 | 0 | 0 | 0 | 0 | 0 | 0 | 0 | 0 | 0 | 0 | 0 | 0 |
| NIV 131 |  | 0 | 0 | 0 | 0 | 0 | 0 | 3 | 0 | 0 | 0 | 0 | 0 | 0 | 0 | 0 | 3 |
| NIV 132 |  | 0 | 0 | 0 | 0 | 3 | 0 | 3 | 0 | 0 | 0 | 0 | 0 | 0 | 0 | 0 | 0 |
| NIV 133 | PHV02 (2 x 10^7^ pfu) Day -14 | 0 | 0 | 0 | 0 | 0 | 0 | 0 | 0 | 0 | 0 | 0 | 0 | 0 | 0 | 0 | 0 |
| NIV 134 |  | 0 | 0 | 0 | 0 | 0 | 0 | 0 | 0 | 0 | 0 | 0 | 0 | 0 | 0 | 0 | 0 |
| NIV 135 |  | 0 | 0 | 0 | 3 | 6 | 13 | 18 | 15 | 3 | 0 | 5 | 0 | 0 | 0 | 0 | 0 |
| NIV 136 |  | 0 | 0 | 0 | 10 | 13 | 13 | 13 | 10 | 13 | 0 | 0 | 0 | 6 | 0 | 3 | 3 |
| NIV 137 |  | 0 | 0 | 0 | 0 | 5 | 0 | 10 | 0 | 3 | 3 | 3 | 3 | 3 | 0 | 0 | 0 |
| NIV 138 |  | 0 | 0 | 5 | 10 | 13 | 10 | 13 | 10 | 10 | 0 | 3 | 0 | 10 | 0 | 0 | 0 |
| NIV 141 | PHV02 (2 x 10^7^ pfu) Day -7 | 0 | 0 | 5 | 0 | 0 | 5 | 5 | 10 | 10 | 5 | 5 | 0 | 0 | 0 | 0 | 0 |
| NIV 142 |  | 0 | 0 | 3 | 0 | 0 | 0 | 0 | 0 | 0 | 0 | 0 | 0 | 0 | 0 | 0 | 0 |
| NIV 143 |  | 0 | 3 | 3 | 3 | 0 | 0 | 0 | 5 | 11 | 3 | 0 | 0 | 3 | 0 | 0 | 0 |
| NIV 144 |  | 0 | 0 | 3 | 3 | 0 | 0 | 0 | 0 | 3 | 8 | 5 | 5 | 6 | 0 | 0 | 0 |
| NIV 145 |  | 0 | 10 | 3 | 6 | 3 | 3 | 3 | 15 | 15 | 18 | 15 | 0 | 3 | 3 | 6 | 0 |
| NIV 146 |  | 0 | 0 | 3 | 0 | 0 | 0 | 0 | 20 | 20 | 10 | 10 | 10 | 11 | 3 | 3 | 0 |
| NiV 139 | rVSV-EBOV Control (2 x 10^7^ pfu) Day -7 | 0 | 3 | 0 | 5 | 5 | 13 | 10 | 10 | 25 |  | | | | | | |
| NiV 140 |  | 0 | 0 | 0 | 0 | 0 | 5 | 15 | 20 | 35 |  | | | | | | |
| NiV 147 |  | 0 | 0 | 0 | 0 | 0 | 5 | 5 | 5 | 35 |  | | | | | | |
| NiV 148 |  | 0 | 5 | 0 | 0 | 0 | 8 | 13 | 13 | 35 |  | | | | | | |

**Supplementary Table 3**. Thoracic radiograph scores, by treatment group and day after Nipah (Bangladesh) IN/IT challenge on Day 0, Studies 1 and 2. Sum of scores for all 6 lung lobes, by animal. 0=no infiltrates; 1= mild, 2=moderate; 3=severe.

| **A. Study 1 Down dosing** | |  |  |  |  |  |  |  |  |  |  |  |
| --- | --- | --- | --- | --- | --- | --- | --- | --- | --- | --- | --- | --- |
| **Monkey** | **Treatment** | D0 | D1 | D3 | D7 | D10 | D14 | D21 | D28 | D35 | D41 |  |
| NiV 123 | PHV02 High dose (1.7 x 10^6^ pfu) | 0 | 0 | **1** | 0 | **0** | **1** | **1** | **1** | **2** | 0 |  |
| NiV 124 |  | 0 | 0 | **2** | **1** | **1** | **1** | 0 | **1** | **1** | **2** |  |
| NiV 125 |  | 0 | **1** | **1** | **2** | **1** | **1** | **1** | **1** | **1** | **1** |  |
| NiV 126 |  | 0 | **2** | **2** | **2** | **2** | **2** | 0 | 0 | 0 | **2** |  |
| NiV 119 | PHV02 Mid dose (1.8 x 10^4^ pfu) | 0 | **2** | **2** | **2** | **2** | **1** | **1** | **1** | **1** | **1** |  |
| NiV 120 |  | 0 | **1** | **1** | 0 | 0 | 0 | 0 | **2** | 0 | **1** |  |
| NiV 121 |  | 0 | **1** | **2** | **1** | **1** | 0 | **1** | 0 | 0 | 0 |  |
| NiV 122 |  | 0 | 0 | 0 | **1** | **1** | 0 | **1** | **1** | 0 | **1** |  |
| NiV 115 | PHV02 Low dose (<6.6 x 10^2^ pfu) | 0 | **3** | **3** | **13** |  |  |  |  |  |  |  |
| NiV 116 |  | 0 | **4** | **6** | **13** |  |  |  |  |  |  |  |
| NiV 117 |  | 0 | **1** | **1** | **1** | **1** | **0** | **1** | **1** | 0 | **1** |  |
| NiV 118 |  | 0 | 0 | **3** | **1** | **1** | **1** | **2** | **1** | **2** | **2** |  |
| NiV 111 | rVSV-EBOV Control (2 x 10^7^ pfu) | 0 | **2** | **2** | **8** |  |  |  |  |  |  |  |
| NiV 112 |  | 0 | **2** | **3** | **7** |  |  |  |  |  |  |  |
| NiV 113 |  | 0 | **0** | **2** | **8** |  |  |  |  |  |  |  |
| NiV 114 |  | 0 | **4** | **7** | **12** |  |  |  |  |  |  |  |
|  |  |  |  |  |  |  |  |  |  |  |  |  |
| **B. Study 2 Time to protection** | |  |  |  |  |  |  |  |  |  |  |  |
| **Monkey** | **Vaccination** | **D0** | **D1** | **D3** | **D7** | **D8** | **D10** | **D14** | **D21** | **D28** | **D35** | **D42** |
| NIV 127 | PHV02 (2 x 10^7^ pfu) Day -21 | 0 | **4** | **5** | **3** | NT* | **3** | **3** | **2** | **1** | **2** | 0 |
| NIV 128 |  | 0 | **1** | **1** | **4** | NT | **3** | **1** | **1** | 0 | **1** | 0 |
| NIV 129 |  | 0 | 0 | 0 | **1** | NT | **1** | 0 | 0 | **1** | 0 | 0 |
| NIV 130 |  | 0 | **3** | **3** | **1** | NT | 0 | 0 | 0 | 0 | **1** | **1** |
| NIV 131 |  | 0 | **1** | 0 | 0 | NT | 0 | 0 | 0 | 0 | 0 | 0 |
| NIV 132 |  | 0 | 0 | 0 | **1** | NT | 0 | 0 | 0 | **1** | **1** | **1** |
| NIV 133 | PHV02 (2 x 10^7^ pfu) Day -14 | 0 | 0 | **2** | 0 | NT | **2** | 0 | 0 | **1** | **2** | 0 |
| NIV 134 |  | 0 | 0 | **1** | 0 | NT | 0 | **1** | **1** | **1** | **1** | **1** |
| NIV 135 |  | 0 | **1** | **1** | **1** | NT | 0 | 0 | **1** | **1** | **1** | 0 |
| NIV 136 |  | 0 | **1** | **2** | **1** | NT | **1** | **1** | **1** | 0 | **2** | **2** |
| NIV 137 |  | 0 | **2** | **4** | **2** | NT | **2** | 0 | **3** | **1** | **1** | n/d |
| NIV 138 |  | 0 | 0 | **2** | **1** | NT | **1** | 0 | 0 | **2** | 0 | 0 |
| NIV 141 | PHV02 (2 x 10^7^ pfu) Day -7 | 0 | 0 | **2** | **2** | NT | **1** | **1** | **1** | **1** | **1** | 0 |
| NIV 142 |  | 0 | **1** | **2** | **1** | NT | **1** | **1** | **1** | **1** | **1** | **1** |
| NIV 143 |  | 0 | **1** | **2** | **3** | NT | **3** | **3** | **1** | **2** | **2** | **2** |
| NIV 144 |  | 0 | 0 | **2** | **2** | NT | **1** | **1** | 0 | 0 | 0 | 0 |
| NIV 145 |  | 0 | 0 | 0 | **1** | NT | **2** | 0 | 0 | 0 | 0 | 0 |
| NIV 146 |  | 0 | **1** | 0 | **1** | NT | **1** | **1** | 0 | 0 | 0 | 0 |
| NIV 139 | rVSV-EBOV Control (2 x 10^7^ pfu) Day -7) | 0 | 0 | 0 | 0 | **18** |  |  |  |  |  |  |
| NIV 140 |  | 0 | 0 | 0 | 0 | **3** |  |  |  |  |  |  |
| NIV 147 |  | 0 | 0 | **1** | **1** | **17** |  |  |  |  |  |  |
| NIV 148 |  | 0 | 0 | **1** | **1** | **2** |  |  |  |  |  |  |
|  |  |  |  |  |  |  |  |  |  |  |  |  |

| *NT=not tested | |  |  |  |  |  |  |  |  |  |  |  |
| --- | --- | --- | --- | --- | --- | --- | --- | --- | --- | --- | --- | --- |
|  |  |  |  |  |  |  |  |  |  |  |  |  |

**Supplementary Table 4.** Study 2 AGMs vaccinated with PHV02 on Days -21, -14, or -7 or with rVSV-EBOV (control) on Days -14 or -7: neutralizing antibody titers by day with respect to challenge, survival, post challenge clinical and lung radiograph score, viremia, and antibody response

| **Monkey** | **Treatment** | **Study Day with respect to challenge (Day 0)** | | | | | | | **Outcome** | **Nipah viremia** | **Peak challenge viremia (copies/mL) [Day]** |  | **Maximum lung radiograph score** | **≥4-fold antibody rise post challenge** |
| --- | --- | --- | --- | --- | --- | --- | --- | --- | --- | --- | --- | --- | --- | --- |
|  |  | **Day -21** | **Day -14** | **Day -7** | **Day -1** | **Day +3** | **Day +7** | **Day +42** |  |  |  | **Maximum clinical score** |  |  |
| NiV 127 | PHV02 (2 x 10^7^ pfu) Day -21 | <5 | <5 | <5 | **10** | **20** | **80** | **320** | Survived | No | ─ | 0 | 5 | Yes |
| NiV 128 |  | <5 | <5 | **10** | **80** | **160** | **160** | **160** | Survived | No | ─ | 0 | 4 | No |
| NiV 129 |  | <5 | <5 | **10** | **80** | **160** | **160** | **160** | Survived | No | ─ | 3 | 1 | No |
| NiV 130 |  | <5 | <5 | **10** | **40** | **320** | **320** | **320** | Survived | No | ─ | 0 | 3 | Yes |
| NiV 131 |  | <5 | <5 | **160** | **320** | **320** | **320** | **640** | Survived | No | ─ | 3 | 1 | No |
| NiV 132 |  | <5 | <5 | **40** | **160** | **320** | **320** | **320** | Survived | No | ─ | 0 | 1 | No |
| NiV 133 | PHV02 (2 x 10^7^ pfu) Day -14 | <5 | <5 | <5 | **80** | **160** | **160** | **160** | Survived | No | ─ | 0 | 2 | No |
| NiV 134 |  | <5 | <5 | <5 | <5 | **80** | **160** | **320** | Survived | No | ─ | 0 | 1 | Yes |
| NiV 135 |  | <5 | <5 | <5 | **5** | **10** | **40** | **40** | Survived | No | ─ | 18 | 1 | Yes |
| NiV 136 |  | <5 | <5 | <5 | <5 | **10** | **40** | **160** | Survived | No | ─ | 13 | 2 | Yes |
| NiV 137 |  | <5 | <5 | <5 | **20** | **40** | **160** | **320** | Survived | No | ─ | 5 | 4 | Yes |
| NiV 138 |  | <5 | <5 | <5 | **20** | **80** | **160** | **320** | Survived | No | ─ | 13 | 2 | Yes |
| NiV 141 | PHV02 (2 x 10^7^ pfu) Day -7 | <5 | <5 | <5 | <5 | **5** | **10** | **160** | Survived | No | ─ | 10 | 2 | Yes |
| NiV 142 |  | <5 | <5 | <5 | **10** | **10** | **80** | **640** | Survived | No | ─ | 3 | 2 | Yes |
| NiV 143 |  | <5 | <5 | <5 | <5 | **10** | **40** | **640** | Survived | No | ─ | 11 | 3 | Yes |
| NiV 144 |  | <5 | <5 | <5 | <5 | <5 | **20** | **320** | Survived | Yes | 2.91 [D+7] | 8 | 2 | Yes |
| NiV 145 |  | <5 | <5 | <5 | **5** | **5** | **40** | **320** | Survived | No | ─ | 18 | 2 | Yes |
| NiV 146 |  | <5 | <5 | <5 | <5 | **5** | **40** | **320** | Survived | No | ─ | 20 | 1 | Yes |
| NiV 139 | rVSV-EBOV Control (2 x 10^7^ pfu) Day -7 | <5 | <5 | <5 | <5 | <5 | <5 | ─ | Died | No | 5.9 [D+7] | 25 | 18 | ─ |
| NiV 140 |  | <5 | <5 | <5 | <5 | <5 | <5 | ─ | Died | Yes | 5.5 [D+7] | 35 | 3 | ─ |
| NiV 147 |  | <5 | <5 | <5 | <5 | <5 | <5 | ─ | Died | Yes | 5.6 [D+7] | 35 | 17 | ─ |
| NiV 148 |  | <5 | <5 | <5 | <5 | <5 | <5 | ─ | Died | Yes | 5.5 [D+7] | 35 | 2 | ─ |

**Supplementary Figure 1**. Study 1 Geometric mean neutralizing antibody titer (95% Confidence interval (CI), High (1.7 x 10^6^ pfu), Mid (1.8 x 10^4^ pfu) and Low (<6.6 x 10^2^ pfu) dose of PHV vaccine Day -14 (14 days after vaccination), Day -1 (pre-challenge) and Day +42 after NiV challenge. The two non-responders in the Low dose group were excluded for analysis. At each time interval, there was no statistical difference between dose groups (p=0.4645, two-way ANOVA).

**Supplementary Figure 2**. Geometric mean (95% CI) clinical scores, AGMs vaccinated in Study 2 with PHV 02 21, 14 or 7 days or rVSV-EBOV (controls) 14 or 7 days before challenge with Nipah (Bangladesh)


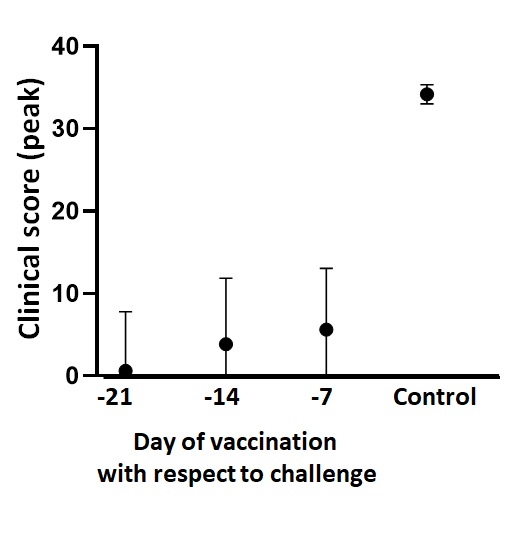


**Supplementary Figure 3.** Linear regression analysis, maximum thoracic radiographic score by pre-challenge neutralizing antibody titer

1. Study 1 Maximum thoracic radiograph score by neutralizing titer pre-challenge (Day -1), animals vaccinated IM with graded doses of PHV02 28 days before IN/IT challenge with Nipah (Bangladesh). Regression analysis without (left panel) and with random noise [± 1.0log2) added to the pre-challenge NTs to assess the impact of measurement error] (right panel)


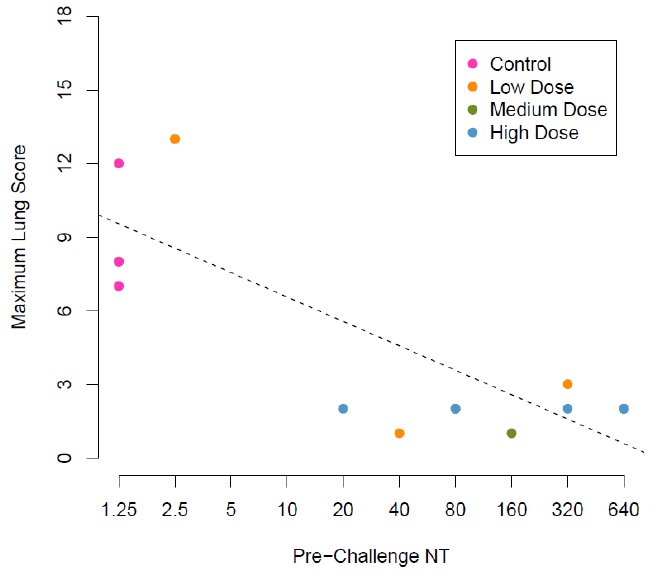

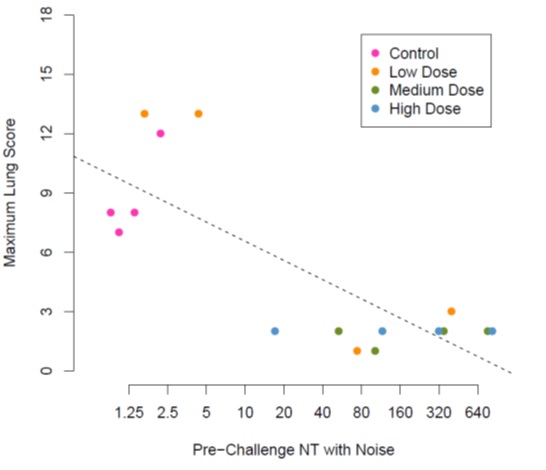


1. Study 2 Maximum thoracic radiograph score by neutralizing titer pre-challenge (Day -1), animals vaccinated IM with PHV02 2 x 10^7^ pfu 21, 14 or 7 days or with rVSV-EBOV 14 or 7 days before IN/IT challenge with Nipah (Bangladesh). Regression analysis without (left panel) and with random noise [± 1.0log2) added to the pre-challenge NTs to assess the impact of measurement error] (right panel)


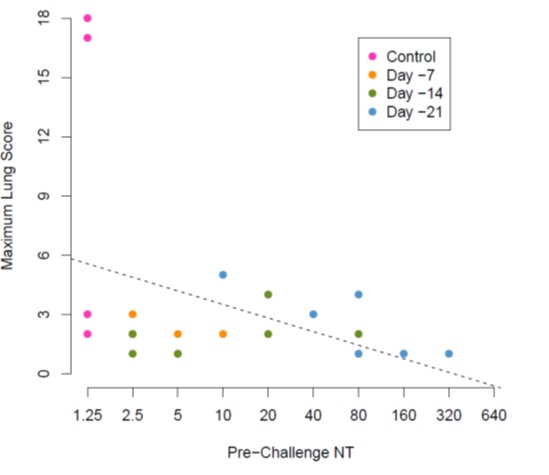

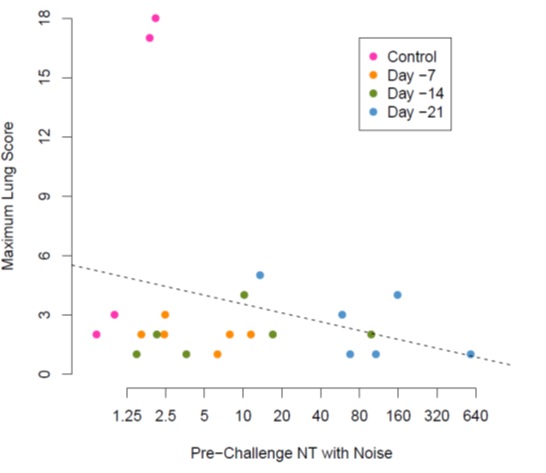


**Supplementary Figure 4**. Reverse cumulative distribution of pre-challenge (Day -1) neutralizing titers survivor animals in Studies 1 and 2 by occurrence of a ≥fourfold increase in titer after NiV challenge. The distributions of titers differ significantly (p=0.0009, log rank test). The probability of having a ≥fourfold rise in antibody after challenge was significantly reduced at a pre-challenge titer of ≥1:40 (p=0.0001, Fisher’s exact test, 2-sided).


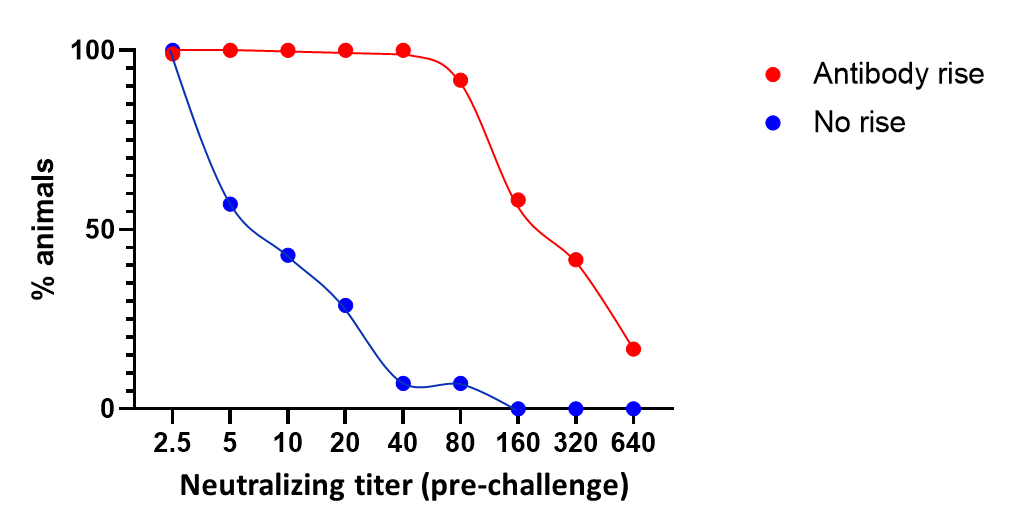


**Supplementary Figure 5.** Study 2 Nipah virus RNA in necropsy tissues. The dashed line indicates the LLOQ of the qRT-PCR

**Supplementary Figure 6.** Expected vs. observed geometric mean fold increase (GMFI) of neutralizing antibody following challenge of AGMs with Nipah (Bangladesh)

Expected increase in neutralizing titer was determined for monkeys following vaccination and compared to the observed increase of vaccinated animals after challenge.

Group A= Day -21 treatment group, GMFI between Day -7 to Day -1 (expected response between 14 and 21 days after vaccination only)

Group B= Day -14 treatment group, GMFI between Day -1 to Day +7 (observed response between 14 and 21 days after vaccination with challenge on day 14)

Group C= Day -14 treatment group, GMFI between Day -7 to Day -1 (expected response between 7 and 14 days after vaccination only)

Group D= Day -7 treatment group, GMFI between Day -1 to Day +7 (observed response between 7 and 14 days after vaccination with challenge on day 7)

| **Groups compared** | **GMFI (95% CI)** | | **P value^1^** |
| --- | --- | --- | --- |
|  | **Expected (vaccination)** | **Observed (Vaccination + challenge)** |  |
| A vs. B | 4.49 (2.60, 7.76) | 10.8 (3.07, 33.06) | 0.2118 |
| C vs. D | 4.00 (0.933, 17.13) | 8.00 (5.05, 12.67) | >0.9999 |

^1^ Unpaired *t* test, two-tailed

**Supplementary Figure 7**. Study 2 Day -1 neutralization titer vs. IgG ELISA, AGMs vaccinated with PHV02 21, 14, or 7 days before NiV challenge.
